# Naturalistic acquisition in an early language classroom

**DOI:** 10.3389/fpsyg.2014.00329

**Published:** 2014-04-17

**Authors:** Anne Dahl, Mila D. Vulchanova

**Affiliations:** Department of Language and Literature, NTNU Norwegian University of Science and TechnologyTrondheim, Norway

**Keywords:** second language acquisition, naturalistic acquisition, input, early-start, classroom

## Abstract

This study investigated whether it is possible to provide naturalistic second language acquisition (SLA) of vocabulary for young learners in a classroom situation without resorting to a classical immersion approach. Participants were 60 first-grade pupils in two Norwegian elementary schools in their first year. The control group followed regular instruction as prescribed by the school curriculum, while the experimental group received increased naturalistic target language input. This entailed extensive use of English by the teacher during English classes, and also during morning meetings and for simple instructions and classroom management throughout the day. Our hypothesis was that it is possible to facilitate naturalistic acquisition through better quality target language exposure within a normal curriculum. The students' English vocabulary knowledge was measured using the Peabody Picture Vocabulary Test, version 4 (PPVT-IV, Dunn and Dunn, 2007a), at the beginning and the end of the first year of school. Findings are that (1) early-start second-language (L2) programs in school do not in themselves guarantee vocabulary development in the first year, (2) a focus on increased exposure to the L2 can lead to a significant increase in receptive vocabulary comprehension in the course of only 8 months, and (3) even with relatively modest input, learners in such an early-start L2 program can display vocabulary acquisition comparable in some respects to that of younger native children matched on vocabulary size. The overall conclusion is that naturalistic vocabulary acquisition is in fact possible in a classroom setting.

## Introduction

Over the past decades, there has been a trend in many countries of lowering starting ages for learning foreign languages, especially English. One reason is globalization and the role of English as an international lingua franca; another is increased knowledge of the benefits of young starting ages for language acquisition. However, the relationship between what we know about language acquisition and what goes on in early language classrooms is not straightforward, and it is not obvious that such classrooms make the best possible use of the learners' young age. A number of studies (e.g., Burstall, 1975; Holmstrand, 1982; Cenoz, 2003; García Lecumberri and Gallardo, 2003; García Mayo, 2003; Lasagabaster and Doiz, 2003; Muñoz, 2006) indicate that an early start in a foreign language does not necessarily make a difference in terms of the pupils' attained competence.

Even though the common assumption that children always acquire languages more easily than adults has been contested (see e.g., Singleton and Ryan, 2004 p. 72 ff. for an overview), the conclusion from findings in research is generally that the earlier one starts acquiring a language before adulthood, the better the chances are of attaining target competence (Johnson and Newport, 1989; Hyltenstam, 1992; DeKeyser, 2000; Hyltenstam and Abrahamsson, 2003; Singleton and Ryan, 2004 ch. 7). This is often attributed to a difference in learning style, as well as maturational constraints related to a sensitive period in language learning (Felix, 1985; Bley-Vroman, 1989; Newport, 1990). Yet little is known about how the factors known to impact on language acquisition interact in the course of development, and what their relative weighting is.

Nikolov (2009) hypothesizes that a possible explanation for the lack of an early-start advantage in previous studies may be that classroom activities employed in that research were better suited to older learners. Quite often the maturational facts in language acquisition link naturally to the learning style differences, namely that younger learners are more likely to employ implicit learning, whereas older learners outperform them on explicit learning (Muñoz, 2006). It then follows that what younger learners need above and beyond all else is exposure to the target language—not explicit instruction and formal training. We know that L2 learners are fully capable of acquiring linguistic knowledge without intentional effort or instruction, and that reading and listening alone can lead to acquisition especially in young learners (cf. Lightbown, 2000; Lightbown et al., 2002). Amount and quality of input are undoubtedly crucial factors in SLA (cf. Hyltenstam, 1992; Gass, 2003), and there is evidence that sensitivity to frequency is relevant for the acquisition of grammatical items (cf. Larsen-Freeman, 1975; Goldschneider and DeKeyser, 2001). Frequency of language items and volume of language exposure have also been demonstrated to influence vocabulary size, at least in L1 acquisition (Hart and Risley, 1995; Childers and Tomasello, 2002; Hoff and Naigles, 2002; Vulchanova et al., 2012), e.g., contributing to learning from distributional cues, a mechanism found in both L1 and L2 acquisition (e.g., Saffran et al., 1996, 1997; Pelucchi et al., 2009).

As Wode (1981, p. 302) points out, “[t]here is no learner on record who learned a language or even part of it without receiving some language input.” Instruction and explicit knowledge may play a role in SLA, specifically in compensating for the limited time and opportunity for exposure in the language classroom (cf. Lightbown, 2000). However, it is likely that explicit instruction is less relevant for young learners, and that cognitive maturity may be necessary in order for explicit forms of instruction to make up for impoverished input (see e.g., DeKeyser, 2000; Muñoz, 2001; DeKeyser and Larson-Hall, 2005; Larson-Hall, 2008). There is thus reason to believe that high-input child SLA contexts are the successful ones, and that both intensity and continuation of exposure are decisive factors (Burstall, 1975; Stern, 1983; Lightbown, 2000; Abello-Contesse et al., 2006; Ruiz-González, 2006; Larson-Hall, 2008: 56).

The crucial question is whether acquisition in early-start L2 classrooms can be significantly improved even with only a limited increase in the amount and density of exposure to English. This can be achieved by giving the language itself a more central place in the English classroom, e.g., in conducting classroom management in English and in prioritizing input-heavy activities such as the teacher reading aloud. In addition, L2 input can be increased also outside of English class by providing classroom management and simple instructions in English throughout the school day. The present study is, to our knowledge, the first study to use such an approach, and to investigate the effect of such increased input on vocabulary acquisition in the context of English as an L2 in Norway.

Norwegian children start learning English systematically in school from age 6. However, the number of teaching hours is low, normally less than one per week (Utdanningsdirektoratet, 2006, 2007), and the English input to which students are exposed is thus necessarily limited. Furthermore, the Norwegian early English classroom typically does not provide very much L2 input, since it largely uses Norwegian as the language of instruction. One reason for this situation may be that English is not an obligatory subject in teacher training in Norway, although most teachers in lower primary school will normally have to teach the subject (cf. Guldal in Trønder-Avisa, 2007). Also, the curriculum and teaching materials commonly used in the classroom reflect a teaching style where the target language is the object, but not the medium of instruction.

Vocabulary acquisition in an L2 has traditionally been associated with rote learning and memorization of words (cf. Kersten, 2010). However, L2 vocabulary can obviously also be acquired from naturalistic input, as is the case in L1. In fact, vocabulary acquisition may not be subject to age effects. While it is the area of language first evident in young children, we all continue to learn new words throughout life. We know that vocabulary is acquired at a fast pace in school (see e.g., Nagy and Herman, 1987; Clark, 1993; Pinker, 1994; Bloom, 2000, 2004; Berman, 2007). On the other hand, vocabulary is an aspect of language for which L1 and L2 acquisition may be assumed to differ. In L1, vocabulary acquisition entails the daunting task of learning concepts and words at once. The L2 learner, on the other hand, will generally have acquired the concepts already. Many theories have been proposed about bilingual vocabulary acquisition, some involving the L1 as a mediator, while others assume a direct link to the concept. Without engaging in a discussion of the extent to which cross-linguistic lexical variation reflects deeper conceptual differences, we assume that L2 vocabulary acquisition, at least at early stages, and at least when the L1 and the L2 represent similar cultures, does to a large extent entail learning the new labels for familiar concepts (see e.g., Singleton, 1999, p. 48; MacWhinney, 2005).

There is thus no reason to believe that neither age nor the presence of an already acquired L1 should have a detrimental effect on vocabulary acquisition, and we should expect that increased exposure to an L2 during the first year of school will lead to naturalistic acquisition and significantly increased vocabulary comprehension. Specifically, it is likely that input alone is particularly beneficial for vocabulary acquisition in young L2 learners. Shintani (2011) explicitly investigated whether input-only instruction may be as effective as production-based instruction for 6–8-year old Japanese learners of English, hypothesizing that mechanisms such as fast mapping are still available at this age. The conclusions of her study are indeed that in this age group, the effect of input-based instruction on vocabulary acquisition is as good as, or better than, that of production-based instruction.

## Method

The present study investigates whether employing a bilingual approach to an otherwise normal Norwegian first-grade English classroom will lead to improved acquisition over 1 year, compared to a standard, i.e., largely native language-based, first grade class. The research questions are whether children in each of the classes improve significantly in vocabulary acquisition over their first year of school, and whether there is a measurable difference in the two groups' vocabulary comprehension at the end of the first grade.

### Conditions

Two different schools were recruited for the experiment. In one school, teachers were told to do nothing out of the ordinary, and to teach English to their first-graders the way they would normally do, with the L1 as the main medium of instruction. In the other school, teachers agreed to use English more extensively with the children in and outside of English class, such as for morning meetings, simple instructions during the day, and reading aloud. However, they were not instructed to avoid the use of the L1; this school's approach to English teaching can thus be said to be bilingually-based.

The two schools were both standard state schools, situated in similar suburban areas in one of Norway's largest towns. The areas from which the schools recruit their pupils are socioeconomically comparable; they are both relatively affluent, with mean incomes slightly above the national average. The ethnic makeup of the two neighborhoods is also comparable, with a low percentage of families with immigrant backgrounds. On the national tests of English for 5th grade in 2008 the two schools scored similarly at or (in the case of the native language-based classroom's school) a little above the average. Thus, there is every reason to believe that these two schools are comparable in terms of student population and quality of teaching, and that they are representative of Norwegian state schools. In addition, a parent questionnaire asked for background information about the children concerning factors such as foreign travel, English-speaking friends and relatives, and input received through media. None of the participants included in the study had extended stays beyond normal vacations abroad, and none had close English-speaking family or other special circumstances which might make their English competence atypical for a Norwegian 6-year-old. Although the parental reports were relatively crude, information was quantified by counting weeks spent outside of Scandinavia and hours per week with English exposure from games and media prior to starting school. This information is summarized for both groups in Table 1.

**Table 1:** **Mean, minimum and maximum values and standard deviations (*SD*) for weeks spent outside of Scandinavia and hours of exposure from media, games and music prior to starting school in the bilingually-based and the native language-based groups**.

	**Weeks outside Scandinavia**		**Exposure from media, games, music**	
	**BB**	**NB**	**BB**	**NB**
Min	0.0	0.0	0.0	0.0
Max	14.0	24.0	11.0	7.0
Mean	4.2	6.4	1.8	1.2
*SD*	3.6	6.8	2.5	1.6

BB, bilingually-based; NB, native language-based.

A Mann-Whitney *U* test found no significant difference for weeks spent outside Scandinavia [*U*_(59)_ = 399.0, *Z* = −0.751, *p* = 0.453], nor on previous exposure to English [*U*_(59)_ = 407.0, *Z* = −0.652, *p* = 0.515]. It thus seems safe to assume that these children's English exposure outside of school was similar.

Children in the two groups were also similar on a number of factors that may potentially influence English acquisition, which will be more closely described in the test materials section.

In each school, three different classes and class teachers participated in the project. In the bilingually-based school, one teacher had the main responsibility for English classes in all groups. In this school, groups were often organized across classes for various subjects, and this teacher was a natural choice for English classes since she was a native speaker of English. However, all class teachers participated in providing input throughout the school day. In each school, one teacher was responsible for recording information on time spent on English, and about activities and materials used, and to report to the researcher. These reports were frequent and relatively informal during the two periods of test sessions in September and May. In the middle of the spring term, both teachers formally reported on the same three questions (time, activities, and materials) in emails to the researcher. Information from both schools indicated that they consistently followed the pattern described below throughout the test period.

The native language-based condition school reported formally spending 30 min a week on English class. They also reported spending a few minutes in morning meetings every day talking about the weather and the names of the days in English, but these meetings were otherwise conducted entirely in Norwegian. The time spent on English in this group was thus of around 45 min per week, which is representative of the average that normal Norwegian schools spend in the first grade. Also representative is the fact that communication during this time took place mainly in Norwegian. Activities in this group included the use of the workbook Junior Scoop 1–2 (Bruskeland and Ranke, 2005) which is intended for use in first grade, and which contains simple activities, including routine instructions, rhymes and songs. Furthermore, teachers reported a number of other English songs used in class.

The bilingually-based group spent about 30–40 min per week on English class. While this school also uses the Scoop series of work- and textbooks, it was not used in this group of children. The teachers instead used various other materials, including simple stories and books, which the teacher read aloud, often with illustrations. Teachers would also spend time talking about pictures or objects. Furthermore, this group spent more time speaking English during morning meeting time; the teacher estimated about 5–10 min per day. While the native language-based group's morning meetings were conducted in Norwegian, with only routine discussion of words for the weather and the days of the week in English, morning meetings in the bilingually-based group were more or less conducted in English on the part of the teacher, while the pupils were free to answer in either language as they wished. Teachers in the bilingually-based school also used English for simple classroom management throughout the day, often with Norwegian translations instantly following, such as the reminder “No running in the corridors—*ikke løpe i gangen*!.”

It is important to point out, then, that the change in the English classroom of the bilingually-based group did not consist of more formal instruction or an increase in teaching hours for English. Time spent on English was a little higher than is normal in Norwegian schools, but with an average of around 70 min per week including morning meetings, it still is a small proportion of the total time spent at school. Furthermore, there was no focus on pupils' production, even though increased L2 production may have been a natural consequence of the increased input. In other words, the change in this school consisted solely of an increased focus on providing target language exposure in a natural context.

### Participants

All parents of students in the relevant first grades were contacted in writing and asked for written consent for their child to participate. Approximately 80% of the parents provided consent in each group. In the bilingually-based group, the total number of volunteers was 59. 10 participants were excluded because they were bilingual, and one because he had participated in another, related study. From the remaining 49 children, 31 were randomly selected for the project by the researcher. The final test group consisted of 17 boys and 14 girls, all monolingual speakers of Norwegian with no known diagnosis which might influence acquisition. In the native-language based group there were 35 volunteers. Three were excluded because of bilingualism, and one because of hearing problems. Two children participated in the pre-test only; one because he was not available during the post-test, the other because he did not want to participate in it. The final test group consisted of 15 boys and 14 girls, all monolingual and with no known diagnosis which may have had consequences for the study.

Mean age at the time of pre-testing was 6;1 in both groups, with no significant difference [*U*_(59)_ = 433.0, *Z* = −0.245, *p* = 0.806]. The project was approved by the Norwegian Social Science Data Services (NSD). Table 2 summarizes descriptive statistics for age and scores on background measures in the two groups.

**Table 2:** **Mean, minimum and maximum values and standard deviations (*SD*) for age, vocabulary, verbal and non-verbal intelligence and memory scores (raw) in the bilingually-based and the native language-based groups**.

	**Age at pretest**		**Norwegian vocabulary**		**English vocabulary (pre-test)**		**English sentences (pre-test)**		**Non-verbal intelligence**		**Verbal intelligenc**		**Memory**	
	**BB**	**NB**	**BB**	**NB**	**BB**	**NB**	**BB**	**NB**	**BB**	**NB**	**BB**	**NB**	**BB**	**NB**
Min	5;6	5;6	97	85	2	3	3	2	12	11	11.0	10.0	40	40.0
Max	6;6	6;5	157	145	56	61	8	10	32	38	24.0	26.0	59	62.0
Mean	6;1	6;1	119.9	113.8	25.4	23.7	5.7	5.2	18.7	17.7	16.6	16.1	46.3	48.0
*SD*	0.027	0.028	14.8	14.6	11.4	13.6	1.5	1.9	4.6	5.2	3.9	4.7	4.6	5.9

BB, bilingually-based; NB, native language-based.

### Test materials

English vocabulary comprehension was tested using Form B of the Peabody Picture Vocabulary Test, Fourth Edition (PPVT™-4). This test measures vocabulary comprehension by means of pictures; the subject hears a word and selects the corresponding picture from a set of four options. This means that issues related to literacy can be avoided, and no L2 production is necessary on the part of the participant. Both these criteria made the test particularly well suited to these young learners, whose level both of literacy and of English was too low, especially in the pre-test, for more comprehensive tests to yield reliable results.

Pre-testing took place within the first 6 weeks of the children's 1st school year. During the pre-test session Norwegian vocabulary comprehension was tested in addition to initial English vocabulary comprehension, using a translated version of Form A of the Peabody Picture Vocabulary Test, Fourth Edition (PPVT™-4). There were no significant differences between the groups on either of these tests.

Post-testing took place during the last 6 weeks of the school year. During this test session, in addition to the post-test of English vocabulary comprehension, visio-spatial working memory was tested using a memory game where the child memorized sets of picture cards which were then turned face down, and was asked to find the pairs in as few attempts as possible. Furthermore, non-verbal intelligence was tested using the Matrices section of the Kaufmann Brief Intelligence Test, Second Edition (KBIT-2), and verbal intelligence using a version of the Riddles section of the KBIT-2 translated into Norwegian. These particular control measures, including L1 vocabulary, were chosen firstly to control for group differences on the outset, and secondly to provide measures that are believed to correlate with L2 acquisition. There are consistent findings in research suggesting that L2 language competence correlates highly with working memory, non-verbal intelligence, and, most importantly, L1 competence and skills (Colledge et al., 2002; Gathercole, 2006; e.g., Sparks et al., 2009; Dale et al., 2010; Hayiou-Thomas et al., 2012; Foyn et al., under revision). No significant differences between the two groups were found on any of the control measures; see Table 3.

**Table 3:** **Mann-Whitney *U*, Z, and *p* for between-groups comparison of vocabulary, verbal and non-verbal intelligence and memory in the bilingually-based and the native language-based groups**.

	**Norwegian vocabulary**	**English vocabulary (pre-test)**	**English sentences (pre-test)**	**Non-verbal intelligence**	**Verbal intelligence**	**Memory**
Mann-Whitney U	352.000	363.000	344.000	333.000	389.500	375.500
*Z*	−1.443	−1.281	−1.402	−1.747	−0.891	−1.098
*p* (two-tailed)	0.149	0.200	0.161	0.081	0.373	0.272

Each test session was conducted at the child's school, during school hours or in the after-school program. Testing took place in a quiet room, with only the child, the researcher, and sometimes an assistant present. Each test session lasted for approximately 1 h. Most children were able to complete test sessions without signs of fatigue; if they did show signs of losing concentration, they were given a short break. Average time between pre- and post-testing was eight months in both groups.

## Results

Because the sample is relatively small (native language-based: *n* = 29, bilingually-based: *n* = 31) and because the data are not normally distributed, data were analyzed with non-parametric tests.

**Figure 1:**
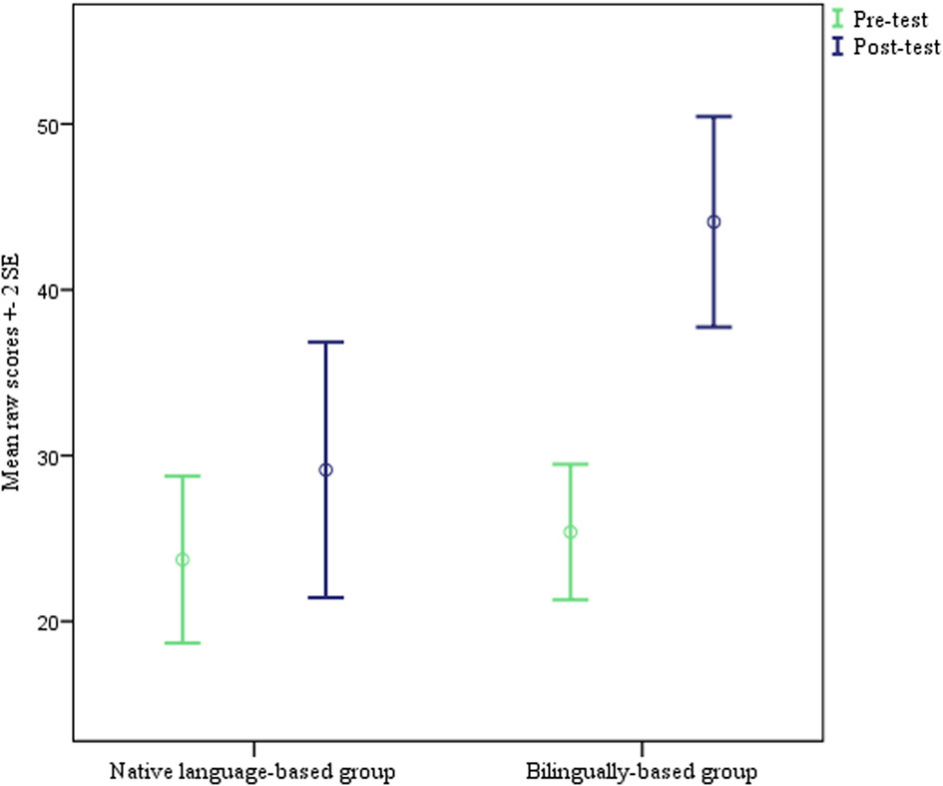
**PPVT-IV receptive vocabulary development, pre-test to post-test**.

Results from the pre-test reveal that the children in general knew very little English when starting school. The mean raw score of the native language-based group was 23.72, which according to the PPVT™-4 manual has an age equivalent for native English speakers of 2;4. The mean raw score in the bilingually-based group was 25.39, with a native age equivalent of 2;5. In short, these Norwegian children demonstrated English comprehension comparable to very young English-speaking children. Both groups' age equivalents are in fact below the chronological age for which the PPVT™-4 is normed, which has a lower bound of 2;6, although they are above the lower bound for age equivalents, which is 2;0. Competence was very similar between the two groups, even though the bilingually-based group did score slightly higher. An independent samples Mann-Whitney *U* test reveals that this difference is not significant [*U*_(59)_ = 363, *Z* = −1.281, *p* = 0.20]. This is confirmed by the PPVT™-4 Manual, which allows raw scores to be converted into growth scale value (GSV) scores. According to the manual (Dunn and Dunn, 2007b, p. 205), between chronological ages 2;6 and 12;0, a GSV point difference of eight is considered significant. The difference between an average score of 23.72 (GSV 84) and one of 25.39 (GSV 85) is only one point, and is thus not significant. Figure 1 illustrates the scores on the pre-test and the post-test in the two groups.

After 8 months, the mean raw score on the PPVT™-4 had increased for both groups; to 29.14 for the native language-based group, and to 44.10 for the bilingually-based group. A Mann-Whitney *U* test found the difference between the two groups at this time to be significant [*U*_(59)_ = 207.5, *Z* = −3.582, *p* < 0.01], and this finding is confirmed by the PPVT™-4 Manual, since the difference between a mean of 29.14 (GSV 89) and a mean of 44.10 (GSV 101) is 12 points.

### Group development

For the repeated-measures test, the Wilcoxon signed ranks test was used. For the native language-based group, the test did not reveal a significant difference between the pre- and post-tests, with *W*_(28)_ = 143.50, *Z* = −1.356, *p* = 0.0875 (one-tailed). This finding is confirmed by GSV scores; the difference between mean pre- and post-test GSV scores is only five points, from 84 to 89 GSV points.

For the bilingually-based group, the median score was significantly different from the pre-test to the post-test with *W*_(30) = 19.50_, Z = −4.479, *p* < 0.01 (one-tailed). Again, the GSV scores confirm the finding, since the difference between mean pre-test (GSV 85) and post-test (GSV 101) score is 16 points, which, according to the PPVT™-4 Manual, is a significant difference (Dunn and Dunn, 2007b, p. 205). The effect size for the bilingually-based group was 0.8, indicating that the change in these pupils' average receptive vocabulary from the beginning to the end of the school year was not only significant but also substantial.

Successful L2 acquisition does not necessarily equal near-nativeness, but comparison to L1 acquisition may nevertheless be useful for purposes of illustration. One measure of the meaningfulness of the development in the bilingually-based group is illustrated in Table 4, which summarizes the results and their age equivalents, as given by the PPVT™-4 manual. Thus, the native language-based group's (non-significant) mean increase in receptive vocabulary translates into an equivalent of only 3 months' development in native English children, from age 2;4 to age 2;7.

**Table 4:** **Age equivalents of pre- and post-test vocabulary scores (raw) in the bilingually-based and the native language-based groups**.

	**PPVT™-4, pre-test**		**PPVT™-4, post-test**	
	**Mean score**	**L1 Age equivalent**	**Mean score**	**L1 Age equivalent**
NB	23.72	2;4	29.14	2;7
BB	25.39	2;5	44.10	3;3

BB, bilingually-based; NB, native language-based.

The mean age equivalent of the bilingually-based group, however, has increased by 10 months in the course of an average time span of 8 months. This means that these L2 learners have, on average, been acquiring new words at a slightly faster rate than the average for children at the same stage of language development, who are acquiring English as their L1. The main difference is that, while this development on average takes place between ages 2;5 and 3;3 in English-speaking children, it took place between mean ages 6;1 and 6;9 in these L2 learners. This is quite an astonishing development, considering that the input to which these children have been exposed is still very limited compared to that of children acquiring their native language. The results thus clearly indicate that there is no inherent problem in the early-start foreign language classroom *per se* preventing it from being successful, at least not with respect to vocabulary development.

### The nature of group vocabulary differences

It is worth looking at group differences for cognates and non-cognates separately, since there may be differences in how the two categories are acquired. Because of the PPVT™-4 discontinuation rule, where for each set of 12 words, testing stops if the participant makes eight or more errors, a few children were tested only on the 12 first words of the test in each session. These 12 words which all participants encountered are listed in Table 5 below, followed by the percentages of children in each group who answered correctly for each word in the post-test, as well as the results of a Mann-Whitney *U* test comparing the two groups' responses for each word. Words that sound similar in the two languages (cognates) are given in bold.

**Table 5:** **Percentages of correct answers and Mann-Whitney U, Z, and p for between-groups comparisons of number of correct answers for cognate and non-cognate words in the bilingually-based and the native language-based groups**.

**Word**	**BB(%)**	**NB(%)**	**Mann-Whitney U (***df*** = **59**)**	***Z***	**Sig (1-tailed)**.
**Cat**	100	100	341	0.000	0.500
**Apple**	100	93.1	310	−1.695	0.045
**Balloon**	100	89.7	321	−0.902	0.184
**Hand**	100	93.1	310	−1.695	0.045
Airplane	32.3	24.1	324	−0.386	0.350
Bird	32.3	27.6	335	−0.139	0.445
**Tree**	96.8	44.8	183.5	−3.516	0.000
Table	90.3	10.3	134.5	−4.304	0.000
**Drinking**	96.8	62.1	208	−3.311	0.001
**Frog**	61.3	55.2	313.5	−0.576	0.283
Money	67.7	44.8	249.5	−1.924	0.027
Umbrella	29	6.9	273	−1.747	0.041

BB, bilingually-based; NB, native language-based.

The words which are successfully identified by virtually all children in both groups are *cat*, *apple*, *balloon*, and *hand*, all of which are phonologically similar to their Norwegian counterparts *katt, eple, ballong*, and *hand*. However, the bilingually-based group scores slightly higher also on these words; for *apple and hand*, the difference is significant. Furthermore, the words *tree* and *drinking* are recognized by virtually all the children in the bilingually-based group, and the difference between the two groups here is significant. These words also sound relatively similar to their Norwegian counterparts *tre* and *drikke*.

However, the children in the bilingually-based group outperform their native language-based group peers also on non-cognates. The percentage of children who correctly identify the words *airplane* and *bird*, whose Norwegian equivalents are *fly* and *fugl* respectively, is slightly higher in the bilingually-based group than in the native language-based group, although the difference is not significant, while the differences for the words *money* (Norwegian *penger*) and *umbrella* (Norwegian *paraply*) are significant, and the word *table* (Norwegian *bord*) is the one with by far the biggest difference in scores. While only 10% of the children in the native language-based group correctly identify this word, it is successfully identified by 90% of the children in the bilingually-based group. Although the number of items is too low to draw firm conclusions, we have an indication that the advantage in the bilingually-based group holds both for cognates and non-cognates.

## Discussion

We see from the above results that English teaching in the native language-based group has had no significant impact on English receptive vocabulary. In other words, the 20+ h out of the 138 h of compulsory English teaching for grades 1–4 which this school is spending in the first grade have not had any measurable effect. We interpret this to mean that the L2 input received through this method of English teaching does not reach the critical threshold needed by children at this age for vocabulary development to take place. Children in both groups had acquired some English vocabulary prior to starting school, possibly through various sources such as computer games, music, TV, and movies. However, this vocabulary was very small for both groups, and included a number of cognates with Norwegian; word learning may have been incidental. The native language-based group's lack of English vocabulary development in the course of 8 months indicates that English exposure outside of school for young children in Norway is not sufficient for systematic acquisition. This further supports Murphy's (2010) argument that spending more time on the L2 in the classroom is especially important for learners who do not have extensive exposure to the target language outside of school.

We have also seen that the advantage in the bilingually-based group holds both for cognates and for non-cognates. Cognate and non-cognate acquisition may be slightly different processes in SLA. For example, Tonzar et al. (2009) show that cognates are acquired more easily than non-cognates both for English and German in Italian learners. Gascoigne (2001) proposes that cognates are in fact retrieved differently from other words in the L2, and that the representations in the two languages in the mental lexicon are partly overlapping. Aukrust (2007) argues that observed differences in whether vocabulary size in the two languages is correlated in bilingual children may be the result of how closely related the languages in question are, and consequently how many cognates there are that can be more or less transferred from one language to the other. Norwegian and English are both Germanic languages, and thus relatively closely related. Although a great portion of the English vocabulary is of Romance origin, basic words are more often Germanic, and a possible hypothesis could have been that the improved performance of the bilingually-based group is mainly a result of cognate comprehension. However, we saw in Table 5 that the children in the bilingually-based group seem to outperform the native language-based group both on words which are cognates in Norwegian and English and on words which are not.

Another question is what the exact problem is for acquisition in the native language-based group. There are two alternative explanations for their lack of measurable development on the PPVT™-4 in the course of 8 months. The first possible explanation is that the vocabulary items tested were not frequent enough in this group's input, while the second is that the words tested in the PPVT™-4 were not present in the input at all. The difference is in whether the input in the native language-based group is best described as generally impoverished, or whether it is just naturally more specialized due to being more limited. The early words in the PPVT™-4 are those expected to be familiar to very young American children, and it is not obvious that these are the same as those emphasized in early Norwegian English classrooms. Since the more limited input of the native language-based group necessarily includes a smaller number of words, it is possible that the children in this group have not happened to come across many of the words of the PPVT™-4, even if they may have acquired other words. This could give them an unfair disadvantage in the test. However, a look at the teaching materials and the activities reported for the native language-based group indicates that the vocabulary in the PPVT™-4 has been used in the classroom. Out of the (very few) words to be found in the native language-based group's work book *Junior Scoop 1–2*, several can be found in the early sets of the PPVT™-4, such as *tree*, *bird*, and *balloon*. Another area of vocabulary which teachers in this group specifically mentioned practicing was body parts, an area also present early on in the PPVT™-4 in words such as *hand* and *neck*. It is obviously impossible to establish whether the children have encountered all the words tested early in the PPVT™-4. Still, there is no reason to believe that the words in the PPVT™-4 are thematically different from those used in the native language-based classroom, and that this has created an unfair advantage for the bilingually-based group in the test.

Since the aim of the present study was to investigate the effect of an increase in English input which actually felt manageable and natural to the teachers in the bilingually-based classroom, the researcher did not interfere with what happened in the classroom, and the structure and nature of input was therefore not carefully controlled. This lack of control potentially raises questions about whether it is the exposure *per se* or specific characteristics of it which have brought about the effect. For example, one of the teachers in the bilingually-based school was in fact a native speaker of English, although she was also completely fluent in Norwegian and taught all other subjects in this language. It is of course conceivable that this teacher's nativeness in itself is what led to increased acquisition in the bilingually-based group. However, this would mean assuming that native speakers are always better teachers of L2s or that language can only successfully be acquired from native-speaker input, which goes against research findings both on L1 development (e.g., Singleton and Newport, 2004) and on second/foreign language teaching (cf. Moussu and Llurda, 2008). The main benefits of the teacher's nativeness, i.e., language proficiency and the confidence to use English extensively, can both be trained also in non-native teachers. It is precisely the conclusion of this paper that teachers should be trained in this.

Another question is whether the native language-based group really is representative of normal Norwegian schools, or whether the lack of acquisition in this group is a result of “poor” teaching. However, as already mentioned, pupils from this school have been previously found to perform above average in national tests in English. Whereas results in these tests may have come from classes taught by teachers other than those in the present study, it is highly unlikely that the school standards of English instruction have dramatically dropped. Furthermore, as with the bilingually-based group, the native language-based teachers knew that their pupils would be tested after 8 months, and were naturally eager for them to do well. If anything, it is likely that they spent more time on English than they would have in a normal year. Finally, and significantly, there is nothing in what the native language-based teachers report that deviates from the stated norms of the curriculum. As with many early-start foreign language programs, nothing in the plans for early English teaching in Norway focuses on extensive input for vocabulary acquisition.

### Conclusion and suggestions for further research

The overall conclusion of the present study is that there is nothing inherent in the classroom situation which prevents successful L2 acquisition in young learners, and that vocabulary can be acquired at a fast rate in an early-start foreign language program. Furthermore, the study indicates that although such acquisition critically depends on input, exposure to the target language need not be unrealistically massive for acquisition to take place.

The PPVT™-4 only investigates receptive vocabulary, and tells us nothing about the productive vocabulary of the children in the study. However, we do know that the two are related, and that receptive vocabulary is important for comprehension, which in turn means that a larger receptive vocabulary allows more advanced input to be processed and understood. In this sense, receptive vocabulary can be assumed to be a predictor for further language acquisition.

A natural next step is to further examine whether such an increase in exposure to the target language has a long-term effect beyond the first year of school, and whether it is also evident in areas other than vocabulary comprehension. Furthermore, more research is needed concerning exactly what kind of input is necessary, including what proficiency level teachers must have attained and whether native input from sources other than the teacher, especially media (i.e., audio and video) can fruitfully be exploited to increase input in early-start second language classrooms.

## Author contributions

Anne Dahl has had main responsibility for the project, including research design, data collection, analysis and interpretation, and drafting and revising the paper. Mila D. Vulchanova has contributed substantially to the conception and design of the research, and to critical revision of the paper for important intellectual content. Both authors have final approvement of the version to be published and agree to be accountable for all aspects of the work.

### Conflict of interest statement

The authors declare that the research was conducted in the absence of any commercial or financial relationships that could be construed as a potential conflict of interest.

## References

[B1] Abello-Contesse C. Chacón-Beltrán R. López-Jiménez M. D. Torreblancaa-López M. M. (2006). Introduction and overview, in Age in L2 Acquisition and Teaching, eds Abello-Contesse C. Chacón-Beltrán R. López-Jiménez M. D. Torreblancaa-López M. M. (Bern: Peter Lang), 7–27

[B2] Aukrust V. G. (2007). Young children acquiring second language vocabulary in preschool group-time: does amount, diversity, and discourse complexity of teacher talk matter? J. Res. Child. Educ. 22, 20 10.1080/02568540709594610

[B3] Berman R. A. (2007). Developing linguistic knowledge and language use across adolescence, in Blackwell Handbook of Language Development, eds Hoff E. Shatz M. (Malden, MA: Blackwell), 347–367

[B4] Bley-Vroman R. (1989). What is the logical problem of foreign language learning?, in Linguistic Perspectives on Second Language Acquisition, eds Gass S. Schachter J. (Cambridge, MA: Cambridge University Press), 41–68

[B5] Bloom P. (2000). How Children Learn the Meanings of Words. Cambridge, MA: MIT Press

[B6] Bloom P. (2004). Myths of word learning, in Weaving a Lexicon, eds Hall D. G. Waxman S. R. (Cambridge, MA: MIT Press), 205–224

[B7] Bruskeland P. A. Ranke C. T. (2005). Junior Scoop 1-2. Oslo: Samlaget

[B8] Burstall C. (1975). French in the primary school: the British experiment. Can. Mod. Lang. Rev. 31, 388–402

[B9] Cenoz J. (2003). The influence of age on the acquisition of English: general proficiency, attitudes and code-mixing, in Age and the Acquisition of English as a Foreign Language, eds García Mayo M. García Lecumberri M. (Clevedon: Multilingual Matters), 77–93

[B10] Childers J. B. Tomasello M. (2002). Two-year-olds learn novel nouns, verbs, and conventional actions from massed or distributed exposures. Dev. Psychol. 36, 11 10.1037/0012-1649.38.6.967 12428708

[B11] Clark E. V. (1993). The Lexicon in Acquisition. Cambridge: Cambridge University Press 10.1017/CBO9780511554377

[B12] Colledge E. Bishop D. V. Koeppen-Schomerus G. Price T. S. Happe F. G. Eley T. C. (2002). The structure of language abilities at 4 years: a twin study. Dev. Psychol. 38, 749–757 10.1037//0012-1649.38.5.749 12220052

[B13] Dale P. S. Harlaar N. Haworth C. M. Plomin R. (2010). Two by two: a twin study of second-language acquisition. Psychol. Sci. 21, 635–640 10.1177/0956797610368060 20483839

[B14] DeKeyser R. M. (2000). The robustness of critical period effects in second language acquisition. Stud. Second Lang. Acquis. 22, 499–533

[B15] DeKeyser R. M. Larson-Hall J. (2005). What does the critical period really mean?, in Handbook of Bilingualism: Psycholinguistic Approaches, eds Kroll J. F. De Groot A. M. B. (Oxford: Oxford University Press).

[B16] Dunn L. M. Dunn D. M. (2007a). Peabody Picture Vocabulary Test. 4th Edn Minneapolis, MN: Pearson Education, Inc

[B17] Dunn L. M. Dunn D. M. (2007b). Peabody Picture Vocabulary Test Manual. Minneapolis, MN: Pearson Education, Inc

[B18] Felix S. W. S. (1985). More evidence on competing cognitive systems. Second Lang. Res. 1, 47–72 10.1177/026765838500100104

[B20] García Lecumberri M. L. Gallardo F. (2003). English FL sounds in school learners of different ages, in Age and the Acquisition of English as a Foreign Language, eds García Mayo M. D. P. García Lecumberri M. L. (Clevedon: Multilingual Matters), 115–135

[B21] García Mayo M. D. P. (2003). Age, length of exposure and grammaticality judgements in the acquisition of English as a foreign language, in Age and the Acquisition of English as a Foreign Language, eds García Lecumberri M. L. García Mayo M. D. P. (Clevedon: Multilingual Matters), 94–114

[B22] Gascoigne C. (2001). Lexical and conceptual representations in more- and less-skilled bilinguals: the role of cognates. Foreign Lang. Ann. 34, 7 10.1111/j.1944-9720.2001.tb02084.x

[B23] Gass S. M. (2003). Input and interaction, in The Handbook of Second Language Acquisition, eds Doughty C. J. Long M. H. (Oxford: Blackwell), 224–255

[B24] Gathercole S. E. (2006). Nonword repetition and word learning: the nature of the relationship. Appl. Psycholinguist. 27, 513–613 10.1017/S014271640606038315257665

[B25] Goldschneider J. M. DeKeyser R. M. (2001). Explaining the “Natural Order of L2 Morpheme Acquisition” in English: a meta-analysis of multiple determinants. Lang. Learn. 51, 1–50 10.1111/1467-9922.00147

[B26] Hart B. Risley T. R. (1995). Meaningful Differences in the Everyday Experience of Young American Children. Baltimore: Brookes

[B27] Hayiou-Thomas M. E. Dale P. S. Plomin R. (2012). The etiology of variation in language skills changes with development: a longitudinal twin study of language from 2 to 12 years. Dev. Sci. 15, 233–249 10.1111/j.1467-7687.2011.01119.x 22356179

[B28] Hoff E. Naigles L. (2002). How children use input to acquire a lexicon. Child Dev. 73, 418–433 10.1111/1467-8624.00415 11949900

[B29] Holmstrand L. S. E. (1982). English in the *Elementary School: Theoretical and Empirical Aspects of the Early Teaching of English as a Foreign Language*. Uppsala: Acta Universitatis Upsaliensis

[B30] Hyltenstam K. (1992). Non-native features of near-native speakers: on the ultimate attainment of childhood L2 learners, in Cognitive Processing in Bilinguals, ed Harris R. J. (Amsterdam: Elsevier Science), 351–368

[B31] Hyltenstam K. Abrahamsson N. (2003). Maturational constraints in SLA, in The Handbook of Second Language Acquisition, eds Doughty C. J. Long C. J. (Oxford: Blackwell), 539–588

[B32] Johnson J. S. Newport E. L. (1989). Critical period effects in second language learning: the influence of maturational state on the acquisition of English as a second language. Cogn. Psychol. 21, 60–99 10.1016/0010-0285(89)90003-0 2920538

[B33] Kersten S. (2010). The *Mental Lexicon* and *Vocabulary Learning: Implications for the Foreign Language Classroom.* Tübingen: Narr

[B34] Larsen-Freeman D. (1975). The acquisition of grammatical morphemes by adult ESL students. TESOL Q. 9, 409–419 10.2307/3585625

[B35] Larson-Hall J. (2008). Weighing the benefits of studying a foreign language at a younger starting age in a minimal input situation. Second Lang. Res. 24, 35–63 10.1177/0267658307082981

[B36] Lasagabaster D. Doiz A. (2003). Maturational constraints on foreign-language written production, in Age and the Acquisition of English as a Foreign Language, eds García Lecumberri M. L. García Mayo M. D. P. (Clevedon: Multilingual Matters), 136–160

[B37] Lightbown P. M. (2000). Classroom SLA research and second language teaching. Appl. Linguist. 21, 431–462 10.1093/applin/21.4.431

[B38] Lightbown P. M. Halter R. White J. Horst M. (2002). Comprehension-based learning: the limits of “Do It Yourself.” Can. Mod. Lang. Rev. 58, 427–464 10.3138/cmlr.58.3.427

[B39] MacWhinney B. (2005). A unified model of language acquisition, in Handbook of Bilingualism: Psycholinguistic Approaches, ed De Groot A. M. B. (Cary, NC: Oxford University Press), 49–67

[B40] Moussu L. Llurda E. (2008). Non-native English-speaking English language teachers: history and research. Lang. Teach. 41, 315–348 10.1017/S0261444808005028

[B41] Muñoz C. (2001). Factores escolares e individuales en el aprendizaje formalde un idioma extranjero, in Estudios de Lingüística. Anexo 1: Tendencias y Líneas de Investigación en Adquisición de Segundas Lenguas, eds Cesteros S. P. García V. S. (Alicante: Universidad de Alicante), 249–270

[B42] Muñoz C. (2006). The BAF Project: research on the effects of age on foreign language acquisition, in Age in L2 Acquisition and Teaching, eds Abello-Contesse C. Chacón-Beltrán R. López-Jiménez M. D. Torreblancaa-López M. M. (Bern: Peter Lang), 81–92

[B43] Murphy V. A. (2010). The relationship between age of learning and type of linguistic exposure in children learning a second language, in Continuum Companion to Second Language Acquisition, ed Macaro E. (London; New York: Continuum International Publishing Group), 158–178

[B44] Nagy W. E. Herman P. A. (1987). Breadth and depth of vocabulary knowledge: Implications for acquisition and instruction, in The Nature of Vocabulary Acquisition, eds Mckeown M. G. Curtis M. E. (Hillsdale, NJ: Erlbaum), 19–36

[B45] Newport E. L. E. (1990). Maturational constraints on language learning. Cogn. Sci. 14, 11–28 10.1207/s15516709cog1401_2

[B46] Nikolov M. (2009). The age factor in context, in The Age Factor and Early Language Learning, ed Nikolov M. (Berlin: Mouton de Gruyter), 1–38

[B47] Pelucchi B. Hay J. F. Saffran J. R. (2009). Statistical learning in a natural language by 8-month-old infants. Child Dev. 80, 674–685 10.1111/j.1467-8624.2009.01290.x 19489896 PMC3883431

[B48] Pinker S. (1994). The Language Instinct: How the Mind Creates Language. New York, NY: Harper Perennial Modern Classics

[B49] Ruiz-González G. (2006). Age effects on single phoneme perception, in Age in L2 Acquisition and Teaching, eds Abello-Contesse C. Chacón-Beltrán R. López-Jiménez M. D. Torreblancaa-López M. M. (Bern: Peter Lang), 155–173

[B50] Saffran J. R. Newport E. L. Aslin R. N. (1996). Word segmentation: the role of distributional cues. J. Mem. Lang. 35, 606–621 10.1006/jmla.1996.0032

[B51] Saffran J. R. Newport E. L. Aslin R. N. Tunick R. A. Barrueco S. (1997). Incidental language learning: listening (and learning) out of the corner of your ear. Psychol. Sci. 8, 4 10.1111/j.1467-9280.1997.tb00690.x10193055

[B52] Shintani N. (2011). A comparative study of the effects of input-based and production-based instruction on vocabulary acquisition by young EFL learners. Lang. Teach. Res. 15, 137–158 10.1177/1362168810388692

[B53] Singleton D. (1999). Exploring the Second Language Mental Lexicon. Cambridge: Cambridge University Press 10.1017/CBO9781139524636

[B54] Singleton D. Ryan L. (2004). Language Acquisition: The Age Factor. Clevedon: Multilingual Matters

[B55] Singleton J. L. Newport E. L. (2004). When learners surpass their models: the acquisition of American Sign Language from inconsistent input. Cogn. Psychol. 49, 370–407 10.1016/j.cogpsych.2004.05.001 15342259

[B56] Sparks R. L. Patton J. O. N. Ganschow L. Humbach N. (2009). Long-term relationships among early first language skills, second language aptitude, second language affect, and later second language proficiency. Appl. Psychol. 30, 725–755 10.1017/S0142716409990099

[B57] Stern H. H. (1983). Fundamental Concepts of Language Teaching. Oxford: Oxford University Press

[B58] Tonzar C. Lotto L. Job R. (2009). L2 vocabulary acquisition in children: effects of learning method and cognate status. Lang. Learn. 59, 23 10.1111/j.1467-9922.2009.00519.x

[B59] Trønder-Avisa (2007). Bekymret for engelskfaget [Online]. Available online at: http://www.t-a.no/nyheter/article176154.ece#.UoEQ2OKmb30 (Accessed November 11, 2013).

[B60] Utdanningsdirektoratet (2006). Knowledge Promotion: Curriculum in English. Available online at: http://www.udir.no

[B61] Utdanningsdirektoratet (2007). Ansvaret for fordeling av skoledager utover året. Available online at: http://www.udir.no

[B62] Vulchanova M. Vulchanov V. Sarzhanova D. Eshuis H. (2012). The role of input in early bilingual lexical development. Lingue e linguaggio 17, 181–198 10.1418/387858789516

[B63] Wode H. (1981). Learning a Second Language: An Integrated View of Language Acquisition. Tuebingen: Narr

